# Obtaining and Characterizing Andean Multi-Floral Propolis Nanoencapsulates in Polymeric Matrices

**DOI:** 10.3390/foods11203153

**Published:** 2022-10-11

**Authors:** Carlos A. Ligarda-Samanez, David Choque-Quispe, Elibet Moscoso-Moscoso, Mary L. Huamán-Carrión, Betsy S. Ramos-Pacheco, Diego E. Peralta-Guevara, Germán De la Cruz, Edgar L. Martínez-Huamán, José C. Arévalo-Quijano, Jenny C. Muñoz-Saenz, Mauricio Muñoz-Melgarejo, Doris M. Muñoz-Saenz, Jimmy Aroni-Huamán

**Affiliations:** 1 Food Nanotechnology Research Laboratory, Universidad Nacional José María Arguedas, Andahuaylas 03701, Peru; 2 Nutraceuticals and Biopolymers Research Group, Universidad Nacional José María Arguedas, Andahuaylas 03701, Peru; 3 Research Group in the Development of Advanced Materials for Water and Food Treatment, Universidad Nacional José María Arguedas, Andahuaylas 03701, Peru; 4 Agroindustrial Engineering, Universidad Nacional José María Arguedas, Andahuaylas 03701, Peru; 5 Water Analysis and Control Research Laboratory, Universidad Nacional José María Arguedas, Andahuaylas 03701, Peru; 6 Agricultural Science Faculty, Universidad Nacional de San Cristobal de Huamanga, Ayacucho 05000, Peru; 7 Department of Education and Humanities, Universidad Nacional José María Arguedas, Andahuaylas 03701, Peru; 8 Department of Human Medicine, Universidad Peruana los Andes, Huancayo 12006, Peru; 9 Social Sciences and Humanities Faculty, Universidad Nacional Enrique Guzman y Valle, Lima 15011, Peru

**Keywords:** nanoencapsulation, propolis, nano spray drying, bioactive compounds, antioxidant capacity

## Abstract

Propolis is a substance with significant anti-inflammatory, anticancer, and antiviral activity, which could be used more efficiently at the nano level as an additive in the food industry. The aim was to obtain and characterize nanoencapsulated multi-floral propolis from the agro-ecological region of Apurimac, Peru. For nanoencapsulation, 5% ethanolic extracts propolis with 0.3% gum arabic and 30% maltodextrin were prepared. Then, the mixtures were dried by nano spraying at 120 °C using the smallest nebulizer. The flavonoid content was between 1.81 and 6.66 mg quercetin/g, the phenolic compounds were between 1.76 and 6.13 mg GAE/g, and a high antioxidant capacity was observed. The results of moisture, water activity, bulk density, color, hygroscopicity, solubility, yield, and encapsulation efficiency were typical of the nano spray drying process. The total organic carbon content was around 24%, heterogeneous spherical particles were observed at nanometer level (between 11.1 and 562.6 nm), with different behaviors in colloidal solution, the thermal gravimetric properties were similar in all the encapsulates, the FTIR and EDS analysis confirmed the encapsulation and the X-ray diffraction showed amorphous characteristics in the obtained material; stability and phenolic compound release studies indicated high values of 8.25–12.50 mg GAE/g between 8 and 12 h, the principal component analysis confirmed that the flora, altitude, and climate of the propolis location influenced the content of bioactive compounds, antioxidant capacity, and other properties studied. The nanoencapsulate from the district of Huancaray was the one with the best results, allowing its future use as a natural ingredient in functional foods. Nevertheless, technological, sensory, and economic studies should still be carried out.

## 1. Introduction

Propolis is a lipophilic resinous material produced by honey bees (*Apis mellifera* L.) and nowadays its use in the food industry is more interesting due to its nutraceutical and functional potential. These properties are attributed to its high content of phenolic compounds, flavonoids, and a high antioxidant capacity; attributes that make it a substance that promotes human health, with important antimicrobial, anti-inflammatory, anticancer, and antiviral activity, which would allow its use as an important natural food additive [1,2,3,4,5,6,7,8]. Nevertheless, their use in food products is still problematic due to their intense aroma, bitter taste, poor thermal stability, low oxidative stability, and insolubility in water [9]. Thus, the nanoencapsulation technique of bioactive compounds is an interesting strategy for the solution to these drawbacks since it could mask their bitter taste and increase the protection of their medicinal properties [10,11,12]. It is known that antioxidants reduce oxidative stress and therefore prevent the onset of degenerative diseases caused by free radicals, so the use of nanoparticles would enhance these benefits [13] and would also improve the bioavailability of the antioxidants present in the natural propolis studied, which could be included in medicines and preferably in functional foods [5].

Nanoencapsulation by spray drying is an alternative method that is gaining strength in the food industry since it is a cost-effective and versatile technique that allows obtaining particles at the nanometer level, which would enhance the beneficial effect on health [14,15,16,17,18]. Recently, diverse polymeric matrices have been used to encapsulate biocompounds, among which gum arabic and maltodextrin stand out due to their better behavior at the nanoscale level [19,20,21,22,23]. Gum arabic is extracted from the trees of *Acacia senegal* L. [24]; which is considered a food biopolymer with a rather complex chemical structure, in which the presence of various carbohydrates predominates, giving it good emulsifying properties and low viscosity in water [25,26], which is why it has been used in nano spray drying processes in recent years [27,28]. Regarding maltodextrin, it is a substance obtained from the hydrolysis of starches from different sources, which, when they have a high dextrose equivalence, are characterized by high solubility in aqueous media, low viscosity, tasteless flavor, and are considered excellent encapsulants that could be acquired at relatively low prices [7,19,29]. As mentioned above, gum arabic and maltodextrin are frequently combined. In recent years, several studies have reported that both wall materials are mixed in different proportions for encapsulating different ingredients used in the food industry, obtaining outstanding results [1,7,19,30]. However, there is also the possibility of using other matrices such as tara gum, tragacanth gum, vinal gum, native potato starch, and glucomannan with lower encapsulation efficiency [1,31,32,33].

Several products from beekeeping are used in the food industry, especially honey and propolis [5]. Up to 850 different chemical compounds have been reported in propolis, among which flavonoids and phenolic acids stand out the most; the physical and chemical properties of propolis vary according to its geographical location, flora, altitude, climate, and type of bee [34] and there are many methods to extract and purify propolis, such as maceration and extraction assisted by high hydrostatic pressure, microwaves, and ultrasound [35]. However, the biggest challenge for its use in functional foods is preserving the bioactive components until the consumption of fortified products [36], there is still little information on propolis encapsulates in food products, although preliminary tests have been carried out on products such as cheese, pudding, refrigerated *Piaractus brachypomus*, and cake [37,38,39]. Of all the products from beekeeping, propolis is a substance that needs special handling because it is a viscous, sticky, and untreatable material. It is obtained from a mixture of secretions produced by bees and resins from the surrounding flora. The origin of propolis directly affects the content of flavonoids, phenolic compounds, antioxidant capacity, and color [40,41]. It is known that propolis from the Andean region is used to prevent and cure various diseases because unique properties are attributed to them due to the particular environment in which they are produced [42], which would allow the bioactive compounds and biological activity to be better than in other regions of the world.

Although propolis is widely studied in the world [5,43], the present research aimed to nanoencapsulate natural propolis from the Andean region of Apurimac, Peru. Adequate proportions of gum arabic and maltodextrin were used, which allowed obtaining encouraging results of physical, chemical, and structural properties, which would allow the potential use of nanoencapsulated multi-floral natural propolis as an additive in the food industry; however, more complementary technological, economic, and sensory studies should be undertaken.

## 2. Materials and Methods

### 2.1. Materials

The raw natural propolis of multi-floral origin were collected during the months of February, March, and April 2022, and were kindly provided by the Association of Beekeepers of the Chumbao Valley. Samples were collected from Huinchos whose flora was eucalyptus *(Eucalyptus globulus),* cypress *(Cupresus macrocarpa)*, pine *(Pinus Radiata),* and chilca *(Baccharis lanceolata)*; in Chaccamarca whose flora consisted of eucalyptus *(Eucalyptus globulus)*, wild turnip *(Brassica napus)*, tasta (*Escallonia myrtilloides)*, alder *(Alnus jorullensis),* and capulí *(Prunus serótina)*; in Huancaray, the flora consisted of eucalyptus *(Eucalyptus globulus*), tasta *(Escallonia myrtilloides)*, alder *(Alnus jorullensis)*, chilca *(Baccharis lanceolata)*, chachacoma *(Escallonia resinosa),* and cypress *(Cupresus macrocarpa)*; and finally Cuncataca whose flora was eucalyptus *(Eucalyptus globulus)*, pine *(Pinus radiata),* and alder *(Alnus jorullensis)*. All the places mentioned above belong to the Province of Andahuaylas, Region Apurimac, Peru.

On the other hand, propolis was also collected from the Pampas River valley, belonging to the province of Chincheros, Apurimac Region, Peru, whose flora consisted of pati (*Eriotheca vargasii*), avocado (*Persea americana*), lemon (*Citrus limón*), mango *(Mangifera indica)*, pacay *(Inga edulis)*, unka (*Myrciantes oreophylla*), peach *(Prunus persica),* and prickly pear *(Opuntia ficus indica)*. The two provinces from which the samples were obtained are considered agroecological, according to the Apurimac Regional Concerted Development Plan 2030. All samples collected were kept in airtight jars and stored at refrigerated temperature until use.

**Figure 1:**
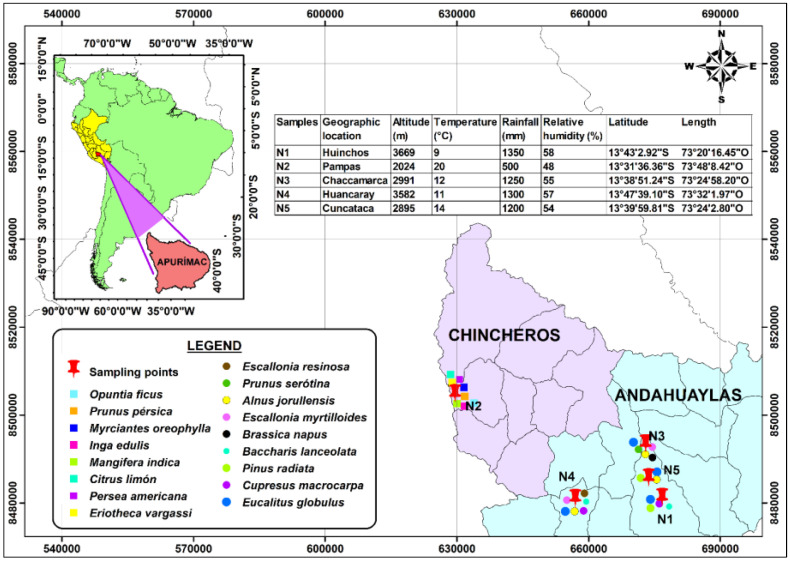
Geographical location of the raw natural multi-floral propolis collection sites.

Figure 1 shows a geographic information system detailing the collection sites of raw natural multi-floral propolis; all other reagents and inputs used in this research were of analytical grade.

### 2.2. Ethanolic Extraction of Propolis

Plastic grids were placed over the honeycombs and under the hive lids to obtain the raw propolis. After three months, the grids were removed and kept at a temperature of 5 °C to facilitate the manual extraction of the natural propolis.

For purification, a total of 15 g of crude propolis was added to 100 mL of 80% ethanol; then, it was shaken for 24 h in a thermo-magnetic shaker M6 (CAT, Ballrechten-Dottingen, Germany). After that time, it was filtered at 0.42 mm in order to eliminate the excess. The ethanolic extract was kept at −15 °C in a LM57SDT refrigerator (LG, Seoul, South Korea) for 10 h. Finally, it was centrifuged at 4500 rpm and 5 °C for 10 min in a refrigerated centrifuge TDL-5M (BIORIDGE, Shanghai, China), and the supernatant obtained was kept at refrigeration temperature until further use.

**Figure 2:**
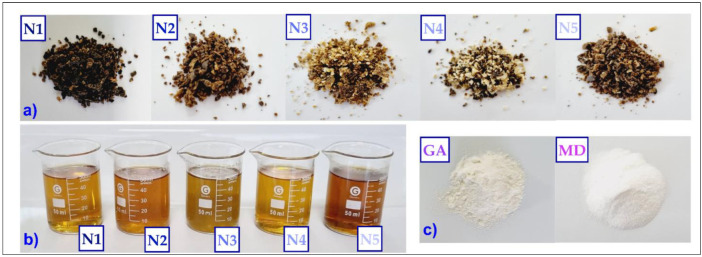
Raw materials and extracts; (**a**) crude propolis (N1, N2, N3, N4, and N5), (**b**) ethanolic extracts propolis (N1, N2, N3, N4, and N5), and (**c**) wall materials, gum arabic (GA) and maltodextrin (MD).

Figure 2a shows the raw natural propolis, Figure 2b shows the ethanolic extracts propolis, and Figure 2c shows the wall materials used.

### 2.3. Nanoencapsulation of Ethanolic Extract of Propolis

**Figure 3:**
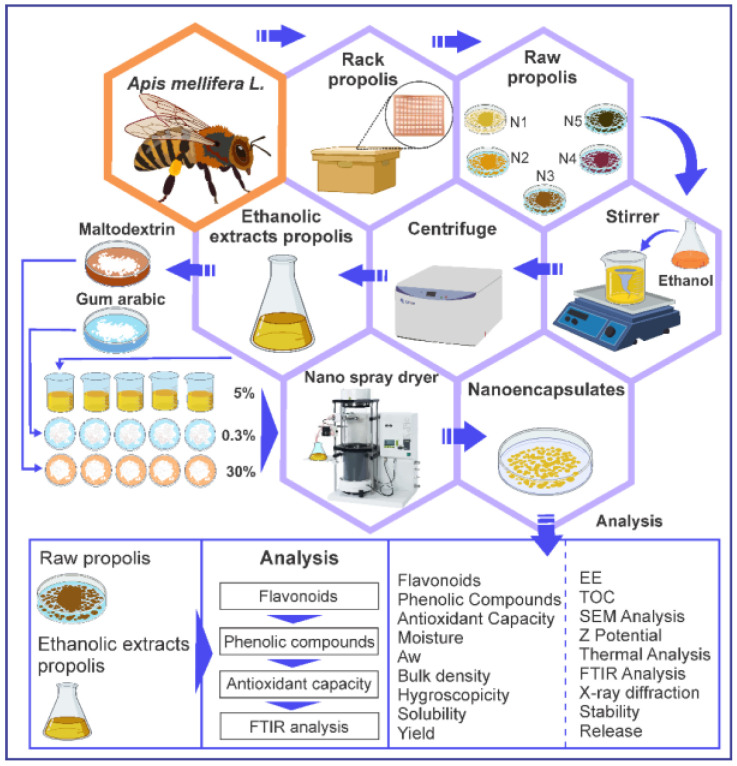
Experimental design for nanoencapsulation.

For the nanoencapsulation of the ethanolic extracts of propolis obtained from the different sites, the experimental design shown in Figure 3 was used, mixing constant amounts of 5% (*w*/*v*) ethanolic extract of propolis, 30% (*w*/*v*) maltodextrin, and 0.3% (*w*/*v*) of gum arabic [1], for a time of 24 h in a thermo-magnetic stirrer M6 (CAT, Ballrechten-Dottingen, Germany). The viscosity was adjusted with distilled water to 8 cps using a DV-E rotational viscometer (Brookfield Engineering Laboratories, Inc., Stoughton, MA, USA), using spindle No. 61.

Finally, encapsulation was performed with a B-90 nano spray dryer (BÜCHI Labortechnik AG, Flawil, Switzerland), at an inlet temperature of 120 °C and outlet temperature of 55 °C, with a gas flow rate of 120 L/h, inlet rate of 30%, pressure of 25 mbar, and 30% spray in the nebulizer. The nanoencapsulates obtained were collected in low-density polyethylene bags and stored in a desiccator until use.

### 2.4. Flavonoid Content

To determine flavonoids in raw propolis, ethanolic extracts of propolis, and nanoencapsulates [44], a calibration curve was previously generated with quercetin (Sigma Aldrich, St. Louis, MI, USA), using concentrations of 0.2, 0.4, 0.6, 0.8, 1, and 1.2 mg/mL; then, 90 µL of sample extract was mixed with 4.81 mL of 80% methanol and 0.1 mL of 5% AlCl_3_ (Sigma Aldrich, St. Louis, MI, USA) in ethanolic solution and left to stand in the absence of light for 10 min. Finally, readings were completed in a Genesys 150 UV spectrophotometer (Thermo Fisher Scientific, Waltham, MA, USA) at a wavelength of 425 nm. Flavonoid content was expressed as mg quercetin per g of sample on a dry basis.

### 2.5. Total Phenolic Compound Content

To determine total phenolic compound content in raw propolis, ethanolic extracts of propolis, and nanoencapsulates, the Folin–Ciocalteu methodology [45] was used, for which a 0.25 N Folin solution (Merck, Darmstadt, Germany) and a 20% Na_2_CO_3_ solution (Spectrum, NB, Canada) were prepared; the calibration curve was generated using gallic acid (Merck, Darmstadt, Germany) at the concentrations of 5, 10, 20, 25, 30, and 35 mg/L.

Finally, 900 µL of a sample (previously extracted in a methanolic solution) were mixed with 150 µL of Na_2_CO_3_, 300 µL of Folin reagent, and 900 µL of ultrapure water. Then, readings were taken in a Genesys 150 UV spectrophotometer (Thermo Fisher Scientific, Waltham, MA, USA) at a wavelength of 755 nm. The results were expressed as mg of gallic acid equivalent (GAE) per g sample on a dry basis.

### 2.6. Antioxidant Capacity by DPPH and ABTS Assays

To determine the antioxidant capacity (AC) in raw propolis, ethanolic extracts of propolis, and nanoencapsulates [46,47] the 2,2 diphenyl-1-picrylhydrazyl DPPH free radical method was used (HIMEDIA, Mumbai, India). The calibration curve was generated with 6-hydroxy-2,5,7,8-tetramethylchroman-2-carboxylic Trolox (Sigma Aldrich, St. Louis, MI, USA). For this purpose, 150 µL of the sample extract (hydrophilic phase) was taken, and 2850 µL of the diluted DPPH stock solution was added to a test tube protected from light. The sample was allowed to react with the diluted DPPH solution and the blank at room temperature of 20 °C. Finally, readings were taken in a Genesys 150 UV spectrophotometer (Thermo Fisher Scientific, Waltham, MA, USA) at 515 nm. The results were expressed in mg of Equivalent of Trolox (ET) per g of sample on a dry basis.

The antioxidant capacity was also determined using the ABTS assay [48,49], for which the reagent 2,2′-azinobis(3-ethylbenzothiazoline-6-sulfonic acid) ABTS (Sigma Aldrich, St. Louis, MI, USA) and potassium persulfate K_2_S_2_O_8_ (Biolab, Argentina) were used. To obtain the ABTS+ radical, 250 µL of K_2_S_2_O_8_ solution at 2.45 mM and 25 mL of ABTS solution at 7 mM were taken, leaving them to stand for 8 h, a Trolox calibration curve (Sigma Aldrich, St. Louis, MI, USA) was also generated. Samples were read at 734 nm in a Genesys 150 UV spectrophotometer (Thermo Fisher Scientific, Waltham, MA, USA), using 0.3 mL of sample extract and 2.7 mL of ABTS+. The results were expressed in mg of Equivalent of Trolox (ET) per g sample on a dry basis.

### 2.7. Moisture, Water Activity (Aw), Bulk Density, and Color in Nanoencapsulates

Water content was determined using the AOAC 950.10 method [50], Aw was determined using the HygroPalm23-AW instrument (Rotronic brand, Bassersdorf, Switzerland), bulk density was calculated by placing a known amount of sample in a 10 mL graduated cylinder, then tapped several times on a flat surface, recording the mass in grams and the volume for the calculation of bulk density. Finally, a CR-5 colorimeter (Konica Minolta, Tokyo, Japan) was used to determine the color parameters.

### 2.8. Hygroscopicity, Solubility, Percentage of Yield, and Encapsulation Efficiency in Nanoencapsulates

In a hermetic container, a total of 1 g of sample was added to 100 mL of a saturated NaCl solution, and it was kept at 25 °C for seven days. After this time, the final weight was recorded, and the following formula was used for the calculation of hygroscopicity.

(1) $$I=(\frac{m_{3-}m_{2}}{m_{2}-{\;m}_{1}})\cdot 100,$$

where I is the mass increment (%), m_1_ is the weight of the empty Petri dish, m_2_ is the weight of the Petri dish + sample, and m_3_ is the weight of the Petri dish + sample after seven days.

The solubility was determined by dissolving 2.5 g of nanoencapsulates in 250 mL of distilled water, then stirred for 5 min until a homogeneous solution was obtained. Subsequently, the supernatant was separated by centrifugation at 5000 rpm for 5 min in a refrigerated centrifuge TDL-5M (BIORIDGE, Shanghai, China). Finally, an aliquot of the supernatant was dried in a forced convection oven FED 115 (BINDER, Tuttlingen, Germany) at 105 °C for 5 h. The following formula was used for the calculation.

(2) $$S=(\frac{m_{2}}{m_{1}})\cdot 100,$$

where S is the percentage of solubility (%), m_1_ is the initial weight of the nanoencapsulate, and m_2_ is the final weight after dissolution.

To determine the percentage of yield, the final weight of the nanoencapsulate was considered concerning the initial weight according to the following formula.

(3) $$Y=(\frac{\mathrm{Pi}}{\mathrm{Pf}})\cdot 100,$$

where Y is the percentage of yield (%), Pi is the initial weight of the materials (g), and Pf is the weight of the nanoencapsulate (g).

Finally, the determination of encapsulation efficiency was performed based on total phenol content [51] by diluting 0.5 g of sample (purified and nanoencapsulated propolis) in 20 mL of 80% methanol and then obtaining the supernatant by centrifugation at 3000 rpm for 15 min in a TDL-5M refrigerated centrifuge (BIORIDGE, Shanghai, China). Gallic acid (Merck, Darmstadt, Germany) was used for the calibration curve, and readings were taken at 755 nm. The following formula was used for the calculation.

(4) $$EE\;\left(\%\right)=\frac{TPCe}{TPCi}\cdot 100,$$

where EE is the encapsulation efficiency (%), TPCe is the total phenol content of the nanoencapsulates, and TPCi is the total phenol content of purified propolis.

### 2.9. Total Organic Carbon (TOC) in Nanoencapsulates

A total carbon analyzer TOC-L CSN-SSM 5000th (Shimadzu, Kyoto, Japan) was used, with an oxygen flow of 150 mL/min, for which the nanoencapsulates were previously homogenized, taking 50 mg in ceramic containers for reading in the equipment mentioned above.

### 2.10. Scanning Electron Microscopy (SEM) and Energy Dispersive X-ray Spectroscopy (EDS) in Nanoencapsulates

For the determination of the morphology and elemental composition of the nanoencapsulates, Prisma E SEM equipment (Thermo Fisher, MA, USA) was used. Before photographing the samples, all the nanoencapsulates were coated with a carbon tape, and then photographs were taken at a magnification of 1000× and an accelerating voltage of 25 kV.

### 2.11. Particle Size and ζ Potential in Nanoencapsulates

In total, 4 mg of nanoencapsulates were dispersed in 5 mL of ultrapure water, then stirred for five minutes at 1000 rpm. Subsequently, ultrasound was applied to the samples for 10 min at room temperature. An aliquot was taken in a capillary to determine the hydrodynamic diameter and size distribution by dynamic light scattering (DLS) using a nano ZLS Z3000 (Nicomp, MA, USA), with a bandwidth of 33 µsec and a scattering angle of 90°; likewise, the same equipment was used to determine the ζ potential, for which 2 mL of the solution were taken in a polystyrene cell, at a wavelength of 632.8 nm for the laser, a scattering angle of −14.14°, and an electric field strength of 5 V/cm [52].

### 2.12. Thermal Analysis of Nanoencapsulates

The analysis was performed on 10 mg of the nanoencapsulates between 20 and 600 °C, at a heating rate of 10 °C per minute, in the presence of N_2_ and using a TGA 550 thermal analyzer (TA Instrument, New Castle, DE, USA), which allowed obtaining the TGA (thermogravimetric analysis) and DTA (differential thermal analysis).

### 2.13. Fourier Transform Infrared Spectroscopy (FTIR)

The identification of the functional groups of the raw propolis and ethanolic extracts of propolis was undertaken using the ATR (attenuated total reflectance) module of the Nicolet IS50 FTIR equipment (ThermoFisher, Waltham, MA, USA), with a resolution of 8 cm^−1^, 32 scans, and using the advanced correction for the diamond crystal, in addition with an angle of incidence of 45 and a refractive index of 1.50.

The nanoencapsulates were measured using the equipment’s transmission module in the mid-IR range from 400 to 4000 cm^−1^, with a resolution of 8 cm^−1^, 32 scans, and using 0.1% KBr tablets.

### 2.14. X-ray Diffraction (XRD) in Nanoencapsulates

X-ray diffraction analysis was performed using a Bruker diffractometer, model D8-Focus (Karlsruhe, Germany), (Cu Kα1 = 1.54 Å) at 40 kV and 40 mA, and a Lynxeye PSD detector.

### 2.15. Stability and Release of Phenolic Compounds in Nanoencapsulates

For the study of the stability and release of phenolic compounds in aqueous solutions, the Folin–Ciocalteu method was used [45], for which solutions of 10 mg/mL of the nanoencapsulates were prepared, which were left at room temperature and in the absence of light for 0, 6, 24, and 48 h, then readings were undertaken in a UV Genesys 150 spectrophotometer (Thermo Fisher Scientific, Waltham, MA, USA) at a wavelength of 755 nm. The results were expressed in mg of gallic acid equivalent per g sample on a dry basis. On the other hand, for the release of phenolic compounds at different pH, the Folin–Ciocalteu methodology was also used, regulating the pH of the solutions to 3, 4, 5, and 6 with buffers of citric acid and 0.1 M dehydrated sodium citrate [7].

### 2.16. Statistical Analysis

One-way analysis of variance (ANOVA) and Tukey’s multiple range test at 95% confidence were used; all results were measured three times. SEM image analysis was performed with the ImageJ program (National Institutes of Health, Bethesda, Rockville, MD, USA), and Origin Pro 2022 software (OriginLab Corporation, Northampton, MA, USA) was used to construct the radar plot, principal component analysis (PCA), and Pearson correlogram.

## 3. Results and Discussion

### 3.1. Flavonoid Content, Phenolic Compounds, and Antioxidant Capacity using ABTS and DPPH

All results are shown in Table 1, where it could be observed that flavonoid contents in raw propolis varied between 2.91 and 10.97 mg quercetin/g, phenolic compounds between 3.02 and 11.50 mg GAE/g, antioxidant capacity by ABTS between 1.29 and 1.83 mg ET/g, and antioxidant capacity by DPPH between 8.98 and 15.80 mg ET/g, with significant differences being observed in most cases (*p* < 5%). As far as ethanolic extracts propolis was concerned, flavonoids varied between 19.79 and 21.28 mg quercetin/g, phenolic compounds between 14.89 and 21.30 mg GAE/g, antioxidant capacity by ABTS between 18.75 and 31. 07 mg ET/g, and antioxidant capacity by DPPH between 81.22 and 106.05 mg ET/g, noting also significant differences in most treatments (*p* < 5%), and a proportional increase in the studied properties of the purified propolis extracts. Similar results were reported for alcoholic extraction methods by maceration and sonication, and in the specific case of the antioxidant capacity reported in the present investigation, higher results were obtained than those reported in the literature [53,54,55,56]. However, higher values were reported in China for propolis extracts obtained by ultrasound [57].

Finally, in the case of nanoencapsulates obtained from purified propolis extract, values were reported for flavonoids between 1.81 and 6.66 mg quercetin/g, for phenolic compounds between 1.76 and 6.13 mg GAE/g, for antioxidant capacity by ABTS between 2.03 and 3. 01 mg ET/g, and for antioxidant capacity by DPPH between 10.36 and 20.07 mg ET/g. Similar values were found in propolis extract encapsulated in gum vinal, gum arabic, and maltodextrin matrices, reporting that the gums were used to contribute to the final content of total phenolic compounds [1]. Overall, the nanoencapsulate N4 showed the highest values in the properties studied, and the higher the flavonoids and phenolic compounds content, the higher the antioxidant capacity [6,7].

The variability of bioactive compounds and antioxidant activity would be attributed to the complexity of the origin of propolis due to the flora, geographical location, altitude, and climate, which affected its chemical composition [58]. In addition, it could be appreciated that the contents of phenolic compounds were higher than those of flavonoids, polyphenols being considered as the main components that confer biological activities to propolis; similar results were reported in Brazilian samples of the brown, green, and red colors [4]. On the other hand, the higher results in the nanoencapsulate from the district of Huancaray (N4) are because this site occupies an intermediate altitude (3582 m) and has a temperate climate where plant species such as eucalyptus (*Eucalyptus globulus*), tasta (*Escallonia myrtilloides*), alder (*Alnus jorullensis*), chilca (*Baccharis lanceolata*), chachacoma (*Escallonia resinosa*), and cypress (*Cupresus macrocarpa*) exist. Furthermore, these results are related to those reported for Peruvian propolis collected in different seasons, in which the diversity of the flora depended on climate change, temperature, humidity, soil type, and location [59].

### 3.2. Characterization of Nanoencapsulates

Table 2 shows all the properties studied; the moisture content of the nanoencapsulates varied between 3.44 and 6.26%, and lower values of between 1.64 and 2.21% were reported for purified propolis microencapsulates [1]; it is recommended that the water content be below 5% in order to increase the shelf life of spray-dried products [60,61,62]. Aw was found between 0.19 and 0.28, which is another important parameter that establishes the amount of free water available to participate in different reaction mechanisms, including enzymatic and non-enzymatic browning; values above 0.6 would indicate greater susceptibility to spoilage [63,64]. Bulk density ranged between 0.47 and 0.48, and the smaller the particle size, the higher the values [62,65]. All treatments presented white colors, and the brightness varied between 91.08 and 92.25, considering color as an essential sensory attribute at the time of acceptance and choice of food products by consumers [6,66]. Color is influenced by the inlet temperature and the concentration of the encapsulants [6].

Regarding the hygroscopicity of purified propolis nanoencapsulates, values between 6.96 and 9.33% were obtained. In a range similar to that determined in microencapsulated propolis (8.4–9.1%) [1], the maximum permissible limit of hygroscopicity for the adequate preservation of dehydrated products is 20%, so all the products obtained in the present investigation comply with not exceeding this critical range [67,68].

The solubility was between 92.72 and 94.44%; this is considered an essential parameter in the powders obtained by atomization since it is an indicator of their behavior during aqueous dissolution [69]. The temperature increase also influences it during the spray drying process [33,70,71]. Finally, the percentage of yield was between 61.26 and 69.40%, quite similar to the mini spray drying equipment in which yields between 60 and 68% were obtained [1].

The encapsulation efficiency was between 11.82 and 28.78%, presenting significant differences in all cases (*p* < 5%). It was observed that nanoencapsulate N4 was the one that obtained the highest percentage, a result that was higher than that reported for nanoencapsulation of purified propolis in gum arabic (1:4), whose value was 21.4% [7]. The high value of the nanoencapsulated N4 would be because the encapsulating materials significantly alter this property and would also be influenced by the interaction between the bioactive compounds, the encapsulant, and the temperature of the drying air used in the nano spray dryer [4]. It was also observed that this drying process allowed to obtain very fine particles and a more uniform size distribution. Nano spray drying is an advanced technological process that should be studied in greater depth for a possible industrial scale-up due to the low EE percentages obtained in this research, which would be related to the fact that this technology uses nebulizers that have a small diameter (4–7 µm) [27], which caused obstructions in the equipment at the time of performing the nanoencapsulation. To solve this problem, some additional operations had to be performed (such as filtration and dilution), which possibly produced a loss of phenolic compounds and an improvement in the solubility of the nanoencapsulates, a hypothesis that would be tested with the particle sizes obtained at the nanometric level [7].

### 3.3. Total Organic Carbon (TOC) in Nanoencapsulates

**Figure 4:**
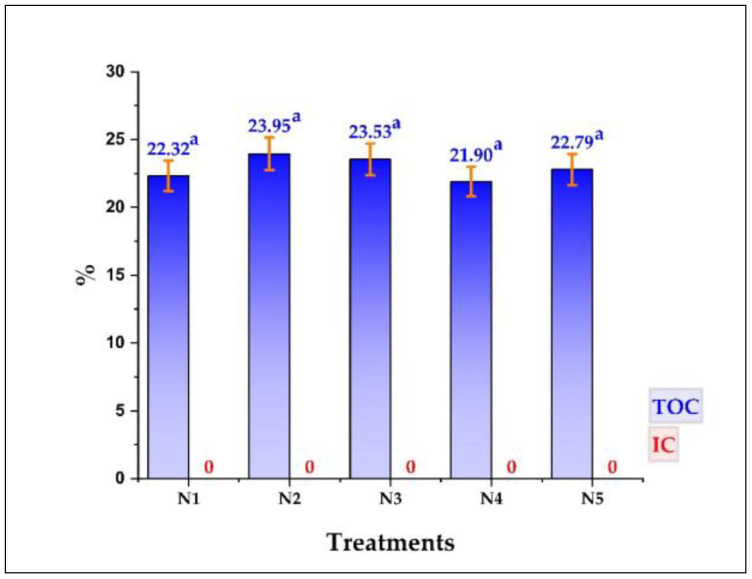
Total organic carbon (TOC) and inorganic carbon (IC) in N1, N2, N3, N4, and N5 nanoencapsulates, equal letters indicate that there are no significant differences, evaluated with the Tukey test at 5% significance.

All the results of TOC and inorganic carbon (IC) determined in the nanoencapsulates are shown in Figure 4, in which it could be observed that no significant differences were found between the nanoencapsulates (*p* > 5%), the data varied between 21.9 and 23.95% for TOC, also noting that there was no presence of IC, since the nanoencapsulates obtained were totally organic. The reported TOC values are because carbon is present in the purified propolis and the wall materials used as part of the structure of proteins, carbohydrates, fats, and fibers [52,72,73]. The obtained TOC data are related to the results of the SEM-EDS surface analysis performed in the present study; in both cases, the presence of organic molecules, typical in spray-dried products in the food industry, would be confirmed [15,19,74,75].

### 3.4. Scanning Electron Microscopy (SEM) and Energy Dispersive X-ray Spectroscopy (EDS) in Nanoencapsulates

**Figure 5:**
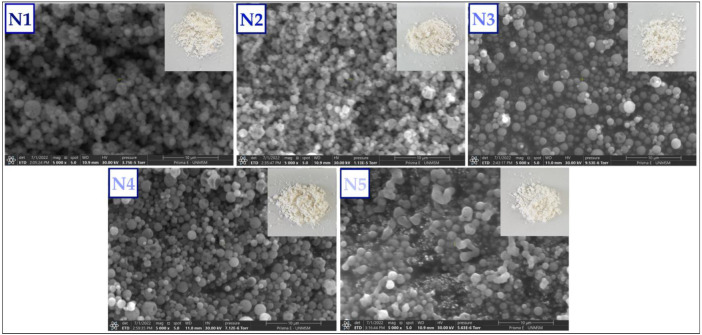
SEM images of propolis nanoencapsulates N1, N2, N3, N4, and N5 at 5000× magnification.

Figure 5 shows the SEM images obtained for the nanoencapsulates, which presented similar spherical shapes, heterogeneous sizes, and smooth surfaces. In some cases, sure holes were observed in the nanoencapsulates, which would be attributed to the increase in evaporation temperature and shrinkage in the spray drying process; similar behavior was reported in other investigations in which maltodextrin and gum arabic were used as wall materials [7,14,60,76].

The continuous homogeneous coating and the absence of indentations on the surface of the particles would indicate that this is a suitable coating matrix, which would allow better protection of the core [77,78]. On the other hand, it was observed that the smaller particles agglomerated on the surface of the larger ones, which is typical in spray drying processes.

**Figure 6:**
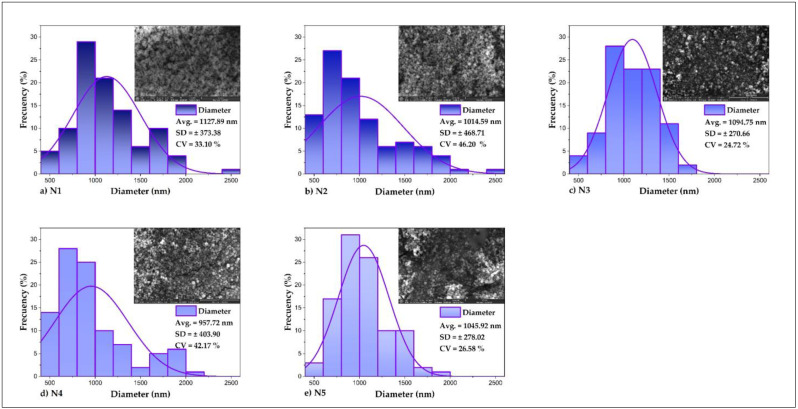
SEM images of nanoencapsulates (**a**) N1, (**b**) N2, (**c**) N3, (**d**) N4, and (**e**) N5, in ImageJ and Origin Pro.

Figure 6 shows the processing of the SEM images in ImageJ and Origin Pro, for which 300 representative measurements of the diameter of the spheres contained in the images of each nanoencapsulate were sampled, obtaining frequency distribution tables and histograms of the nanoparticle diameters, which showed that the sizes mainly were distributed between the range of 500–1000 nm, which coincides with the operating parameters of the B-90 nano spray drying equipment [29,79,80]. Furthermore, average values of the diameter of the encapsulates were reported, which were logically higher than those determined by DLS since there is a greater dispersion of data because they are samples contained in each image obtained by SEM.

Table 3 shows the results of the surface chemical characterization of the nanoencapsulates, which was determined through the EDS module of the SEM. The presence of carbon and oxygen atoms is mostly observed, whose contents varied between 50.4 and 80.3% and between 19.7 and 49.6%, respectively, being the highest carbon atomic percentage in nanoencapsulate N4, which would allow us to presume that the encapsulation was better in this treatment. The SEM-EDS analysis, in general, was carried out to confirm the encapsulation of the purified propolis, which would be related to the presence of C and O, the main chemical elements in the polymeric matrices used to develop the encapsulation [81,82].

### 3.5. Particle Size and ζ Potential in Nanoencapsulates

The results of particle size and ζ potential are shown in Table 4. It could be seen that the size of the nanoencapsulates by NICOMP distribution varied between 11.1 and 562.6 nm, and the Gaussian distribution ranged between 198.1 and 266.7 nm. Moreover, a heterogeneous data dispersion was observed in nanoencapsulates N1 and N5 because of the presence of two peaks in the NICOMP distribution. That would happen due to the agglomeration of the particles due to electrostatic and chemical interactions typical of the nano spray drying processes, further influenced by structural changes in the encapsulates, which, when solubilized in water and atomized, give rise to nanocapsules of different sizes, rich in amino acids, lipids, and carbohydrates [33,83,84].

The ζ potential could vary between ± 100 mV; in the present investigation, the results were between -36.66 and 7.21 mV. This property allowed us to know the stability of the colloidal solutions due to the charge on the surface of the nanoencapsulates; the values obtained for N3 and N5 would indicate a maximum tendency to agglomeration and precipitation; in the case of N2, slight stability and few agglomerates were observed, finally for N1 and N4 moderate stability and no presence of agglomerates were observed [85].

### 3.6. Thermal Analysis in Nanoencapsulates

**Figure 7:**
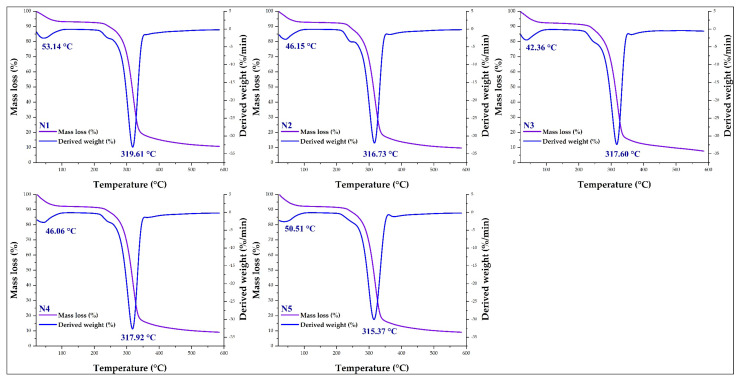
TGA and DTA curves of the nanoencapsulates (N1, N2, N3, N4, and N5).

The nanoencapsulates had similar thermogravimetric behavior. Figure 7 shows the TGA and DTA curves in which similar mass losses could be observed, for temperature ranges from 20 to 600 °C, in which two events were appreciated. The first occurred between 42.36 and 53.14 °C with a mass loss between 4.13 and 4.15%, mainly attributed to the rupture of hydrogen bridges and consequent elimination of water and low-molecular-weight volatile compounds [6,86]. The second event occurred between 315.35 and 319.6 °C, with losses in weight between 52.21 and 54.95%, mainly due to the incineration of proteins, lipids, carbohydrates, and other organic components. Above these temperatures, the other compounds of the encapsulated matrix would be lost until finally reaching residues [74,76,82,87]. The decomposition of free polyphenols and amino acids would occur above 200 °C due to the interaction of phenolic compounds with the polymeric encapsulation matrices [6,88].

### 3.7. Fourier Transform Infrared Spectroscopy (FTIR)

**Figure 8:**
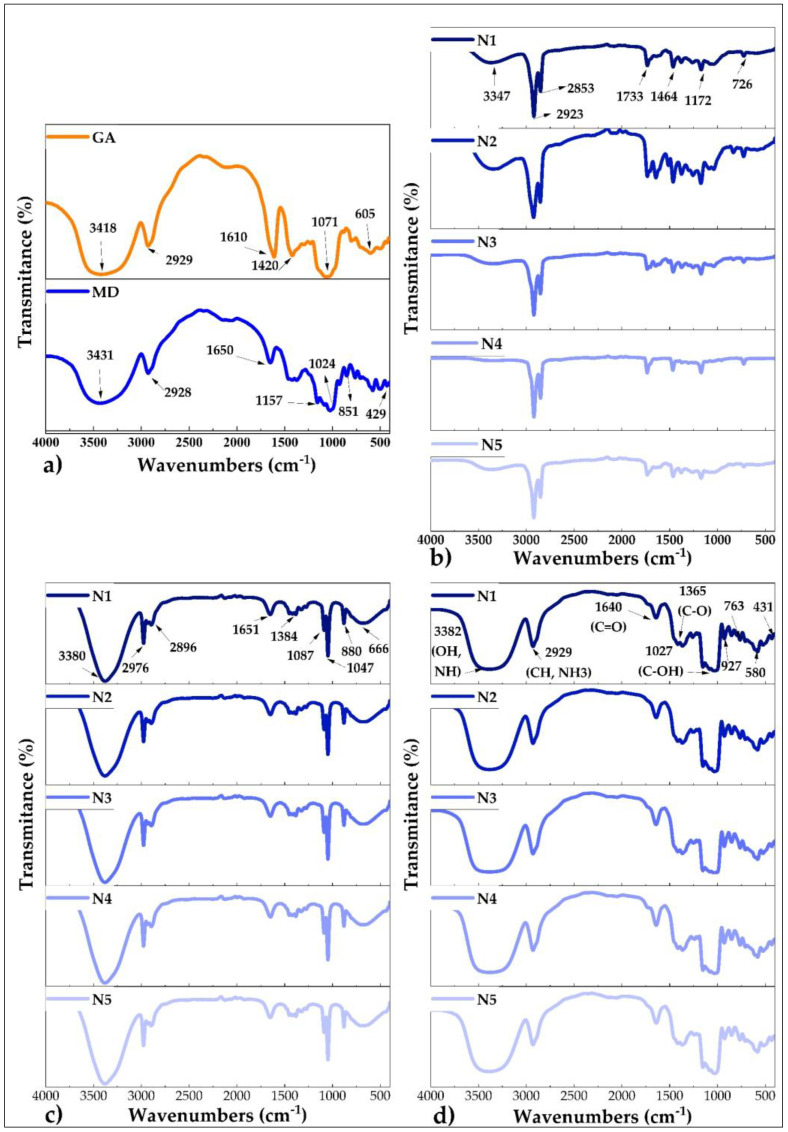
FTIR spectra; (**a**) gum arabic (GA) and maltodextrin (MD); (**b**) raw propolis; (**c**) ethanolic extracts propolis; and (**d**) nanoencapsulates.

Figure 8 shows the spectra obtained by FTIR for the matrices, raw propolis, ethanolic extracts propolis, and nanoencapsulated propolis. It could be seen that the voltage bands are similar for N1, N2, N3, N4, and N5 (Figure 8b–d), showing that the geographic location where the propolis was obtained did not influence the chemical groups of the encapsulates. On the other hand, Figure 8a shows the spectra of the matrices, showing that these affected the functional groups of the atomized products, contributing several chemical groups to the final product (Figure 8d). The FTIR analysis confirmed the nanoencapsulation of propolis due to the molecular structure of the studied material [89], using an approved methodology for FTIR interpretation [90]. Strong stretching tension bands between 3347 and 3382 cm^−1^ were also observed in all the materials, which would correspond to the hydroxyl (OH) and amino (NH) group [4,91], attributed to the possible presence of phenolic compounds, glucides, polypeptides, and water [92,93]. The wave number of 2929 cm^−1^ present in all the encapsulates would be linked to the asymmetric stretching of the chemical groups CH and NH_3_, due to the presence of amino acids and carboxylic acid; the 1640 cm^−1^ voltage band would indicate the existence of carbonyl and ketone functional groups, associated with the presence of fats, polyphenols, flavonoids, and modifications of the aromatic ring in the compounds [94,95], the wave numbers between 1027 and 1365 cm^−1^, would be related to the presence of ether, ester, alcohol, and carboxylic acid chemical groups in the propolis extracts obtained, which in turn are associated with the presence of phenolic compounds and various flavonoids [96,97]; the spectral region between 763 and 927 cm^−1^ would correspond to the C-H group of the aromatic ring present in polyphenols [98], while the peaks between 431 and 580 cm^−1^ would be related to structural modifications of the aromatic rings of the nanoencapsulates [99]. The aforementioned coincides with reports for various bee propolis extracts that were encapsulated [3,4,6].

### 3.8. X-ray Diffraction (XRD) in Nanoencapsulates

**Figure 9:**
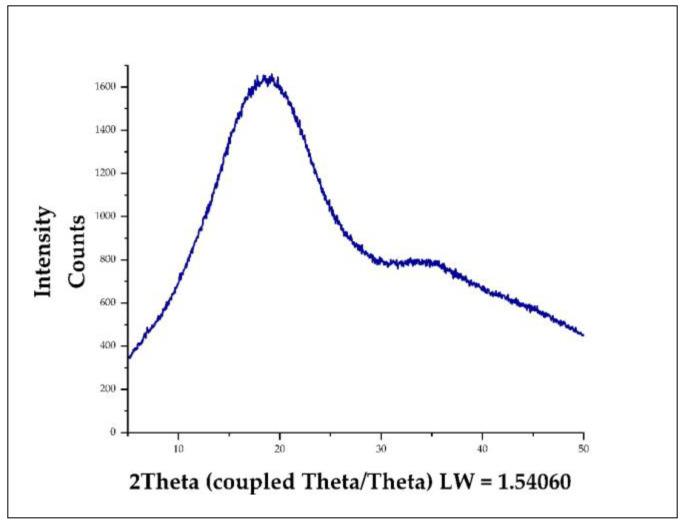
X-ray diffraction pattern of all nanoencapsulates.

Figure 9 shows the X-ray diffraction pattern obtained for the five nanoencapsulates, which would indicate a very low degree of crystallinity in these nanoencapsulates, observing a quite evident peak near 20°, followed by slight diffraction between 30 and 40° that could be due to the particle size and the irregularity of the nanoencapsulates, while the rest of the signal confers amorphous characteristics to this type of product. Those mentioned above would indicate that the purified propolis extracts could have been molecularly dispersed in the wall materials or that the proportion used to perform the spray drying process was insufficient to alter the material’s properties [4,6].

### 3.9. Stability and Release of Phenolic Compounds in Nanoencapsulates

**Figure 10:**
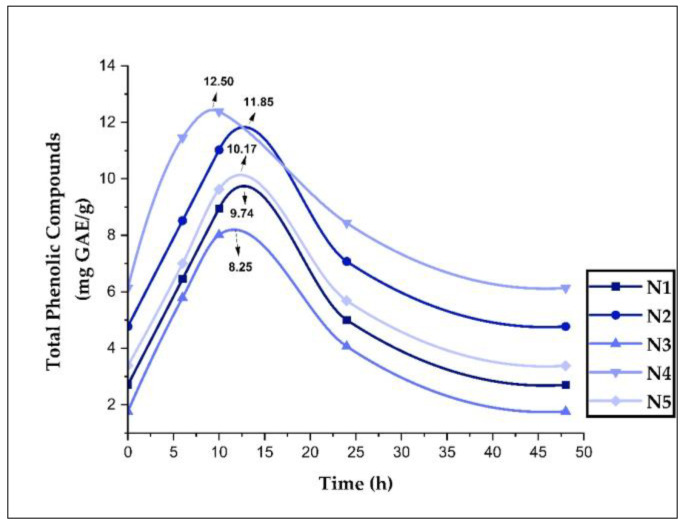
Stability and release of phenolic compounds in aqueous solutions in nanoencapsulates (N1, N2, N3, N4, and N5).

The stability and release of polyphenols in aqueous solutions were tested since the nanoencapsulates would be used in the food industry as natural water-soluble additives. Figure 10 shows that the release profiles were similar in all treatments. It was also noted that the highest number of phenolic compounds released was between 8 and 12 h (8.25–12.50 mg GAE/g), with the highest result in the sample from the Huancaray district (N4). After 24 h, it was observed that the values dropped almost to the initial levels, and it was also noted that at 48 h, the levels were even lower. The kinetic study developed in this research would be beneficial in determining the possible applications of nanoencapsulates. It would also indicate a possible behavior at the gastric and intestinal level when foods enriched with these nanoencapsulates are consumed [7,100].

In the case of the release of phenolic compounds at different pH, no changes were observed for pH 3, 4, 5, and 6; therefore, no release kinetics of the nanoencapsulated compounds could be reported. This behavior coincided with that reported for encapsulated propolis from Brazil [7].

### 3.10. Overview of the Results Obtained in Nanoencapsulates

**Figure 11:**
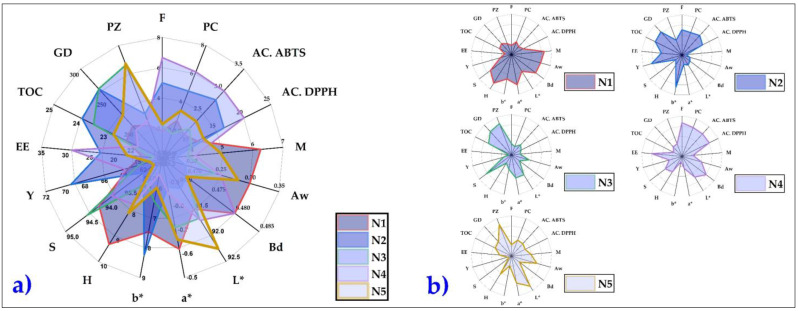
Radar plot of all nanoencapsulated properties (N1, N2, N3, N4, and N5); (**a**) all nanoencapsulates superimposed on top of each other and (**b**) all independent nanoencapsulates.

For the choice of Figure 11, some concepts of data visualization were used for effective communication of information [101]. In this figure, a spider web diagram was shown with all the summarized results of the physical, chemical, and structural properties studied, in which it could be clearly seen that the nanoencapsulated N4 showed the highest values for flavonoids, phenolic compounds, and antioxidant capacity. In addition, N4 showed the best physical, chemical, and structural properties studied, observing that they are within the parameters requested in products obtained by nano spray drying [16,17,21,23,102]. Based on the above and considering the data analysis and graphic visualization, the N4 treatment would be chosen as the best. However, more complex technological, economic, and sensory studies should still be carried out, allowing its potential use as a functional ingredient in the food industry.

**Figure 12:**
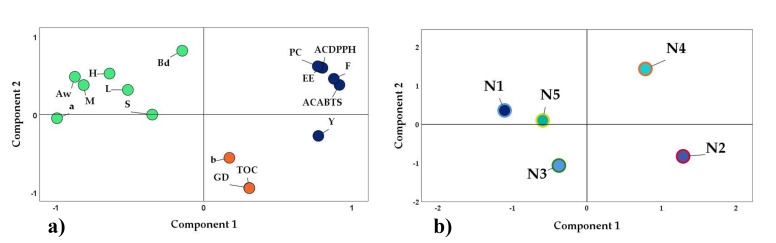
Principal component analysis: (**a**) properties studied and (**b**) nanoencapsulates.

To better understand the findings of this research, a principal component analysis (PCA) [103] was performed on the physical, chemical, and structural properties studied in the nanoencapsulates. In Figure 12a, it can be observed that three groups were formed. The first group in blue was formed by phenolic compounds (PC), flavonoids (F), antioxidant capacity (DPPH and ABTS), encapsulation efficiency (EE), and yield (Y), properties that would be related to the N4 treatment, as observed in Figure 12b. On the other hand, the second group in green color would relate the variables of moisture (M), water activity (Aw), hygroscopicity (H), bulk density (Bd), solubility, chroma a, and L, which are considered necessary during the storage of spray-dried products.

Finally, a third group was observed in orange color confirmed by the particle size of the Gaussian distribution (GD), chroma b, and total organic carbon (TOC). This last parameter confirmed the encapsulation using polymeric matrices such as gum arabic and maltodextrin. These results were related to the data obtained through surface characterization by EDS and FTIR. In the case of treatments N1, N2, N3, N4, and N5, Figure 12b corroborates the significant differences between the chosen sampling sites, which were characterized by having a particular flora, altitude, and climate, which determined these quite marked differences.

**Figure 13:**
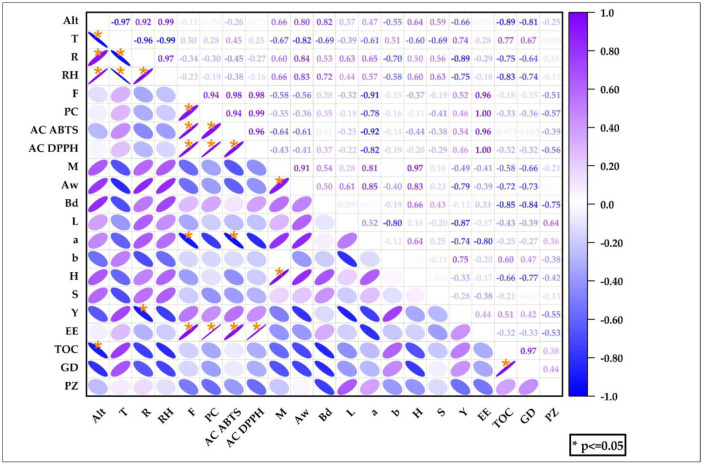
Heat map of climatic conditions and properties studied.

Figure 13 shows the Pearson correlation between climatic conditions and the properties studied; it was observed that altitude (Alt) correlated positively with rainfall (R), temperature (T), and relative humidity (RH) in the study sites; it was also observed that the higher the content of phenolic compounds and flavonoids, the higher the antioxidant capacity and encapsulation efficiency, the total organic carbon also correlated positively with particle size and the same happened between the humidity of the nanoencapsulates with hygroscopicity and Aw. In relation to climatic conditions and the properties studied, it was observed that altitude correlated negatively with TOC, as did rainfall and encapsulation yield. It was observed that sampling locations have an impact on the chemical composition and bio-functional properties of nanoencapsulated propolis [58,104].

## 4. Conclusions

The content of flavonoids, phenolic compounds, and antioxidant capacity of raw propolis, ethanolic extracts propolis, and nanoencapsulates were high. Typical values for the nano spray drying process were observed for moisture, Aw, bulk density, color, hygroscopicity, solubility, yield, and encapsulation efficiency. Instrumental characterization was also performed, confirming the core’s encapsulation in the wall materials, spherical shapes in nanometers, different behavior in colloidal solution, typical functional groups, and amorphous characteristics in the material were observed. The nanoencapsulate N4 presented high values in bioactive compounds and antioxidant capacity; the principal component analysis and Pearson’s correlogram confirmed that climatic conditions influenced the properties studied.

The results obtained on nanoencapsulates allow establishing their potential as an additive in the food industry; however, one of the limitations of the research is the technological, sensory, and economic studies that still need to be carried out for their use, preferably in the enrichment of functional foods.

## Figures and Tables

**Table 1:** Flavonoid content, phenolic compounds, and antioxidant capacity by DPPH and ABTS.

	Flavonoids (mg Quercetin/g)		Phenolic Compounds (mg GAE/g)		Antioxidant Capacity ABTS (mg ET/g)		Antioxidant Capacity DPPH (mg ET/g)	
	$\bar{x}+$ SD	*	$\bar{x}+$ SD	*	$\bar{x}+$ SD	*	$\bar{x}+$ SD	*
Raw propolis								
N1	2.91 ± 0.04	a	3.40 ± 0.02	a	1.30 ± 0.01	a	11.59 ± 0.04	a
N2	9.31 ± 0.03	b	9.66 ± 1.92	b	1.74 ± 0.01	b	13.70 ± 0.19	b
N3	2.92 ± 0.03	a	3.02 ± 0.03	a	1.29 ± 0.01	a	8.98 ± 0.17	c
N4	10.97 ± 0.06	c	11.50 ± 0.01	b	1.83 ± 0.01	b	15.80 ± 0.04	d
N5	3.12 ± 0.03	d	5.29 ± 0.10	a	1.36 ± 0.01	b	12.09 ± 0.03	e
Ethanolic extracts propolis								
N1	13.79 ± 0.25	a	19.09 ± 0.01	a	20.07 ± 0.34	a	94.11 ± 0.18	a
N2	20.24 ± 0.07	b	20.97 ± 0.01	b	25.52 ± 0.16	b	95.58 ± 0.21	b
N3	13.84 ± 0.07	a	14.89 ± 0.08	c	18.75 ± 0.25	c	81.28 ± 0.66	c
N4	21.28 ± 0.10	c	21.30 ± 0.01	d	31.07 ± 0.80	d	106.05 ± 0.27	d
N5	19.3 ± 0.05	d	19.53 ± 0.01	e	22.01 ± 0.31	e	81.22 ± 0.21	c
Nanoencapsulates								
N1	1.81 ± 0.03	a	2.70 ± 0.01	a	2.03 ± 0.11	a	11.46 ± 0.33	a
N2	5.00 ± 0.07	b	4.77 ± 0.01	b	2.81 ± 0.05	b	16.35 ± 0.57	b
N3	2.23 ± 0.04	c	1.76 ± 0.04	c	2.17 ± 0.07	ac	10.36 ± 0.41	a
N4	6.66 ± 0.07	d	6.13 ± 0.01	d	3.01 ± 0.04	d	20.07 ± 1.26	c
N5	2.26 ± 0.04	c	3.38 ± 0.04	e	2.33 ± 0.05	c	12.55 ± 0.21	d

Where N1, N2, N3, N4, and N5 are the geographic location from which the propolis was obtained; $\bar{x}$, arithmetic mean; SD, standard deviation. * Evaluated by Tukey test at 95% confidence, different letters indicate significant differences per column.

**Table 2:** Physical and chemical properties of nanoencapsulates.

	N1		N2		N3		N4		N5	
Property	$\bar{x}+$ SD	*	$\bar{x}+$ SD	*	$\bar{x}+$ SD	*	$\bar{x}+$ SD	*	$\bar{x}+$ SD	*
Moisture (%)	6.26 ± 0.53	a	3.44 ± 0.53	b	3.84 ± 0.52	b	4.10 ± 0.21	bc	4.93 ± 0.19	c
Aw	0.30 ± 0.00	a	0.19 ± 0.53	b	0.24 ± 0.00	c	0.25 ± 0.00	c	0.28 ± 0.01	d
Bulk density	0.48 ± 0.00	a	0.47 ± 0.53	a	0.47 ± 0.00	a	0.48 ± 0.01	a	0.47 ± 0.00	a
L *	91.48 ± 0.04	a	91.08 ± 0.53	b	91.66 ± 0.01	c	91.70 ± 0.01	c	92.25 ± 0.01	d
a *	−0.62 ± 0.01	a	−0.88 ± 0.53	b	−0.71 ± 0.01	c	0.85 ± 0.01	d	−0.66 ± 0.01	e
b *	7.46 ± 0.33	a	8.24 ± 0.53	b	6.44 ± 0.04	c	5.54 ± 0.01	d	5.98 ± 0.02	e
Hygroscopicity (%)	9.33 ± 0.09	a	7.17 ± 0.53	b	6.96b ± 0.27	c	7.78 ± 0.10	cd	8.12 ± 0.39	d
Solubility (%)	94.13 ± 0.02	a	92.98 ± 0.53	ab	94.44 ± 0.02	b	93.79 ± 0.01	ab	92.72 ± 1.34	a
Yield (%)	63.43 ± 1.38	a	69.40 ± 0.53	b	62.44 ± 3.57	a	63.88 ± 2.67	ab	61.26 ± 1.96	a
EE (%)	14.16 ± 0.06	a	22.77 ± 0.53	b	11.82 ± 0.20	c	28.78 ± 0.05	d	17.32 ± 0.08	e

Where N1, N2, N3, N4, and N5 are the nanoencapsulates; Aw, water activity; L * luminosity (0 = black y 100 = white); a * chrome (+a = red, −a = green); b * chrome (+b = yellow y − b = blue); EE, efficiency of encapsulation; $\bar{x}$, arithmetic mean; SD, standard deviation. * Evaluated by Tukey test at 95% confidence, different letters indicate significant difference per row.

**Table 3:** Surface analysis of nanoencapsulates by EDS.

	Element	Atomic %	Atomic % Error	Weight %	Weight % Error
N1	C	73.6	0.2	67.7	0.2
	O	26.4	0.2	32.3	0.3
N2	C	50.4	0.2	43.3	0.1
	O	49.6	0.3	56.7	0.3
N3	C	55.1	0.2	47.9	0.1
	O	44.9	0.2	52.1	0.3
N4	C	77.3	0.2	71.8	0.2
	O	22.7	0.2	28.2	0.3
N5	C	80.3	0.2	75.3	0.2
	O	19.7	0.2	24.7	0.3

Where N1, N2, N3, N4, and N5 are nanoencapsulates; C, carbon; and O, oxygen.

**Table 4:** Particle size and ζ potential.

Treatments	NICOMP Distribution			Gaussian Distribution			ζ Potential (mV)
	Peak	Size (nm)	%	$\bar{x}$	SD	CV (%)	
N1	1	123.4	48.6	204.4	129	63.11	−36.66
	2	562.6	51.4				
N2	1	217.1	100	266.1	158.9	59.71	−28.41
N3	1	228.8	100	266.7	149.9	56.21	6.29
N4	1	188.2	100	198.1	78.3	39.53	−33.45
N5	1	11.1	0.6	222.4	106.3	47.80	7.21
	2	194.4	99.4				

Where N1, N2, N3, N4, and N5 are the purified propolis nanoencapsulates.

## Data Availability

The data presented in this study are available in this same article.
